# How accurate is ceftriaxone at predicting susceptibility of enterobacterales isolates to oral higher-generation cephalosporins?

**DOI:** 10.1128/aac.01387-24

**Published:** 2024-12-31

**Authors:** Kimberly C. Claeys, Patricia J. Simner, Tsigereda Tekle, Anthony D. Harris, Emily Jacobs, Sara E. Cosgrove, Pranita D. Tamma

**Affiliations:** 1 Department of Pharmacy Science and Health Outcomes Research, University of Maryland School of Pharmacy 15513 , Baltimore, Maryland, USA; 2 Department of Medicine, Johns Hopkins University School of Medicine 1500 , Baltimore, Maryland, USA; 3 Department of Pathology, Johns Hopkins University School of Medicine 1500 , Baltimore, Maryland, USA; 4 Department of Epidemiology and Public Health, University of Maryland School of Medicine 12264 , Baltimore, Maryland, USA; 5 Department of Pediatrics, Johns Hopkins University School of Medicine 1500 , Baltimore, Maryland, USA; University of Pittsburgh School of Medicine, Pittsburgh, Pennsylvania, USA

**Keywords:** ceftriaxone, cefdinir, cefpodoxime, cefixime, cefuroxime

## Abstract

The reliability of ceftriaxone for inferring susceptibility to higher-generation oral cephalosporins is unknown. Overall, ceftriaxone susceptibility predicted susceptibility to cefuroxime (89%), cefdinir (86%), cefpodoxime (90%), and cefixime (94%) based on disk diffusion results for 409 consecutive Enterobacterales bloodstream isolates from unique patients. Susceptibility percentages to the four oral cephalosporins ranged from 92% to 99% when limited to *Escherichia coli*, *Klebsiella pneumoniae, Klebsiella oxytoca, or Proteus mirabilis* isolates susceptible to ceftriaxone.

## INTRODUCTION

There is a growing body of evidence supporting early transition to oral antibiotic therapy to complete treatment courses for gram-negative bloodstream infections (GN-BSI) (1–3). Most available data focus on oral fluoroquinolones and trimethoprim/sulfamethoxazole (TMP/SMX) due to their high oral bioavailability (4–6). Although antimicrobial susceptibility testing (AST) results for these agents are generally available to clinicians, toxicities associated with fluoroquinolones (e.g., *Clostridioides difficile* infections, tendonitis, mental status changes, and prolonged QT intervals) and TMP/SMX (e.g., hyperkalemia, hypersensitivity syndromes, and cytopenias), and increasing resistance tempers enthusiasm for these agents (7). Oral beta-lactams are generally well-tolerated but underutilized for a variety of reasons, including lack of AST result availability, wide ranges in oral bioavailability across agents, unclear dosing regimens to optimize pharmacokinetic/pharmacodynamic (PK/PD) efficacy targets, and limited clinical effectiveness data for the treatment of GN-BSI (8).

The Clinical and Laboratory Standards Institute (CLSI) suggests cefazolin (intravenous first-generation cephalosporin) susceptibility be used as a proxy for susceptibility to specific oral cephalosporin agents such as cephalexin (oral first-generation cephalosporin), cefuroxime (oral second-generation cephalosporin), cefdinir (oral third-generation cephalosporin), and cefpodoxime (oral third-generation cephalosporin) (9). However, when an Enterobacterales isolate is *not* susceptible to cefazolin, but is susceptible to ceftriaxone, it is unknown whether higher-generation oral cephalosporin agents retain activity against the isolate as clinical microbiology laboratories generally do not routinely test susceptibility to higher generation oral cephalosporins. We sought to investigate if ceftriaxone susceptibility is an accurate surrogate for susceptibility to commonly prescribed higher-generation oral cephalosporins (i.e., cefuroxime, cefdinir, cefpodoxime, and cefixime) for Enterobacterales isolates to assist clinicians with antibiotic decision making.

### Microbiological methods

All consecutive (non-duplicate) Enterobacterales bloodstream isolates from unique patients processed at the Johns Hopkins Hospital Clinical Microbiology Laboratory from 1 August 2023 until 31 December 2023 were included. Salmonella species were excluded as susceptibility criteria for oral cephalosporins against Salmonella species are not available in the CLSI M-100 document (9). Bacterial species were identified using matrix-assisted laser-desorption ionization time-of-flight mass spectrometry (Bruker Daltonics Inc., Billerica, MA). Fresh bacterial isolates were plated on BBL 150-mm Mueller Hinton agar plates (Becton Dickinson Diagnostics, Sparks, MD). Cefazolin (30 µg), ceftriaxone (30 µg), cefuroxime (30 µg), cefdinir (5 µg), cefpodoxime (10 µg), and cefixime (5 µg) disks (Thermo Fisher Scientific, Waltham, MA) were placed on the same plate using an Oxoid Disc dispenser (Thermo Fisher Scientific). Plates were incubated for 16–18 h. Zone diameters were interpreted by applying susceptibility criteria in accordance with CLSI recommendations (9). Weekly quality control was performed using *E. coli* ATCC 25922.

### Analytic approach

To address the question, “If an Enterobacterales isolate is susceptible to ceftriaxone what is the probability that the isolate will be susceptible to a higher generation oral cephalosporin?” we analyzed data by simple proportions. An identical analysis limited to Enterobacterales isolates resistant to cefazolin but susceptible to ceftriaxone was conducted. It was determined a priori that categorical agreement [i.e., the same categorical interpretation between ceftriaxone and each oral cephalosporin (i.e., both susceptible, both intermediate, and both resistant)] (10) was less relevant as a clinician would be unlikely to prescribe a higher generation oral cephalosporin if an Enterobacterales isolate was not susceptible (i.e., intermediate or resistant) to ceftriaxone in the absence of targeted susceptibility results for the oral cephalosporin of interest .

Furthermore, in the CLSI M-100 Appendix B, there are several organism-antibiotic combinations for which there are concerns for potential intrinsic resistance, defined as expected resistance in wild-type populations of the bacterial species (9). When intrinsic resistance is a concern, the recommendation is to report isolates “as resistant, regardless of measured zone size” (9). Specific to the current study, intrinsic resistance is concerning for cefazolin and several Enterobacterales species expected to produce AmpC enzymes (e.g., *Enterobacter cloacae* complex, *Citrobacter freundii*, and *Klebsiella aerogenes*). Therefore, the above analysis was repeated and limited to four common organisms where excessive AmpC production is typically not a concern (i.e., *Escherichia coli*, *Klebsiella pneumoniae*, *Klebsiella oxytoca*, and *Proteus mirabilis*). This additional analysis also enabled the exclusion of some specific organism-antibiotic combinations where there may be limitations with testing accuracy (e.g., false susceptibility of *Morganella morganii* to cefpodoxime) (9).

### Analysis of the full cohort

A total of 409 Enterobacterales clinical isolates were identified. *Escherichia coli* (200, 49%) was the most common isolate. The distribution of species is presented in Table 1. Across the 409 isolates, 147 (36%) isolates were susceptible to cefazolin and 312 (76%) isolates were susceptible to ceftriaxone. Of the 147 cefazolin-susceptible Enterobacterales isolates, cefuroxime, cefdinir, cefpodoxime, and cefazolin susceptibilities were as follows: 90%, 100%, 100%, and 96%, respectively, confirming the CLSI’s recommendation that cefazolin susceptibility can be used to infer higher generation oral cephalosporin susceptibility. Of concern, 14 (11%) of cefazolin-susceptible isolates were not susceptible to cefuroxime. No clear species-specific trends were observed; however, eight (57%) of these isolates were *Serratia marcescens*. Of the 312 ceftriaxone-susceptible Enterobacterales isolates, susceptibility to cefuroxime, cefdinir, cefpodoxime, and cefixime were as follows: 89%, 86%, 90%, and 94%.

**TABLE 1:** Percentage of ceftriaxone-susceptible Enterobacterales isolates also susceptible to oral higher generation oral cephalosporins by species

Species	Total number of isolates (% total)	Number of cefazolin-susceptible isolates (%)	Number of ceftriaxone-susceptible isolates (%)	Analysis limited to ceftriaxone-susceptible isolates			
				Cefuroxime susceptible (n, %)	Cefdinir susceptible (n, %)	Cefpodoxime susceptible (n, %)	Cefixime susceptible (n, %)
*Citrobacter freundii*	5 (1%)	0 (0%)	3 (60%)	2 (67%)	3 (100%)	2 (67%)	2 (67%)
*Citrobacter koseri*	2 (<1%)	1 (50%)	2 (100%)	2 (100%)	2 (100%)	1 (50%)	2 (100%)
*Enterobacter cloacae* complex	34 (8%)	10 (29%)	24 (71%)	21 (88%)	12 (50%)	19 (79%)	20 (83%)
*Escherichia coli*	200 (49%)	72 (36%)	152 (76%)	146 (92%)	145 (95%)	141 (93%)	148 (97%)
*Klebsiella aerogenes*	6 (1%)	2 (33%)	4 (68%)	4 (100%)	1 (25%)	3 (75%)	3 (75%)
*Klebsiella oxytoca*	7 (2%)	3 (43%)	5 (71%)	5 (100%)	5 (100%)	5 (100%)	5 (100%)
*Klebsiella pneumoniae*	105 (26%)	39 (37%)	80 (76%)	73 (91%)	78 (98%)	72 (93%)	79 (99%)
*Morganella morganii*	5 (1%)	2 (40%)	4 (80%)	--	1 (25%)	3 (75%)	1 (25%)
*Pantoea agglomerans*	2 (<1%)	1 (50%)	2 (100%)	2 (100%)	2 (100%)	2 (100%)	2 (100%)
*Proteus mirabilis*	23 (6%)	9 (39%)	20 (87%)	20 (100%)	19 (95%)	19 (95%)	19 (95%)
*Providencia stuartii*	2 (<1%)	0 (0%)	1 (50%)	1 (100%)	0 (0%)	1 (100%)	1 (100%)
*Serratia marcescens*	18 (4%)	8 (44%)	15 (83%)	0 (0%)	3 (20%)	13 (87%)	14 (93%)
**Overall susceptibility**	**409** (**100%**)	**147** (**36%**)	**312** (**76%**)	**278** (**89%**)	**270** (**86%**)	**281** (**90%**)	**296** (**94%**)
**Overall susceptibility limited to organisms unlikely to produce AmpC enzymes^*a*^**	**335 (100%)**	**123** (**37%**)	**256** (**77%**)	**243** (**95%**)	**246** (**96%**)	**236** (**92%**)	**250** (**98%**)

^*a*^ Includes *E. coli*, *K. pneumoniae*, *K. oxytoca*, and *P. mirabilis.*

The analysis was repeated and limited to cefazolin-resistant Enterobacterales. There was a total of 185 cefazolin-resistant Enterobacterales isolates. Among these isolates, 88 were cefazolin-resistant but ceftriaxone susceptible; susceptibilities to oral cephalosporins were as follows: cefuroxime (79, 90%), cefixime (81, 92%), cefpodoxime (62, 71%), and cefdinir (77, 88%).

### Analysis limited to organisms unlikely to produce AmpC enzymes

The above analysis was repeated and limited to *E. coli*, *K. pneumoniae, K. oxytoca*, and *P. mirabilis* isolates (*n* = 335), given their low propensity for AmpC production (11). Of the 152 ceftriaxone-susceptible *E. coli* isolates, susceptibility to cefuroxime, cefdinir, cefpodoxime, and cefixime was 92%, 95%, 93%, and 97%, respectively. Susceptibilities of the 80 ceftriaxone-susceptible *K. pneumoniae* isolates were similarly high ranging from 91% to 99% across the four oral cephalosporins (Table 1). Susceptibilities to *Proteus mirabilis* (*n* = 20) and *Klebsiella oxytoca* (*n* = 5) ranged from 95% to 100% when ceftriaxone susceptibility was demonstrated.

## Discussion

Overall, our findings suggest that susceptibility to ceftriaxone serves as a reasonable surrogate to infer susceptibility to commonly prescribed higher-generation oral cephalosporins for Enterobacterales when targeted AST results are not available for these agents. More specifically, ceftriaxone susceptibility served as an accurate surrogate for 89%, 86%, 90%, and 94% of isolates tested for cefuroxime, cefdinir, cefpodoxime, and cefixime, respectively. *E. coli, K. pneumoniae*, *K. oxytoca*, and *P. mirabilis* collectively comprised 82% of isolates in our cohort. For these four species, ceftriaxone susceptibility as a proxy for susceptibility to oral cephalosporins was higher—ranging from 92% to 99% across all tested higher-generation oral cephalosporins. Few reports in the published literature investigate the role of ceftriaxone susceptibility as a surrogate for oral cephalosporins. A previous study reported that ceftriaxone susceptibility accurately predicts susceptibility to cefpodoxime 96% of the time, when evaluating *E. coli*, *Klebsiella* species, and *Proteus* species isolates (12).

Importantly, our findings indicate knowledge that an Enterobacterales isolate is resistant to cefazolin without knowledge of ceftriaxone susceptibility results should not be used to assume susceptibility to higher-generation oral cephalosporins. For isolates in this category (i.e., cefazolin-resistant) susceptibility to higher-generation oral cephalosporins ranged from only 71% to 92%. Moreover, although not the primary focus of this work, we found that about 10% of Enterobacterales isolates that are cefazolin susceptible are not susceptible to cefuroxime, raising concerns about the accuracy of cefazolin susceptibility as a surrogate for cefuroxime susceptibility, which needs to be investigated further in future studies.

Ceftriaxone susceptibility to infer susceptibility to higher generation oral cephalosporins was lower for *Enterobacter cloacae* complex, *Klebsiella aerogenes*, and *Citrobacter freundii* isolates in our study. These species are all at moderate risk of clinically significant AmpC production, suggesting that perhaps even basal amounts of AmpC enzymes may be sufficient to hydrolyze higher-generation oral cephalosporins (11). These findings underscore the importance of avoiding higher-generation oral cephalosporins for the treatment of GN-BSI at moderate risk for AmpC production (13). Fortunately, the most common species causing GN-BSI (i.e., *E. coli*, *K. pneumoniae, K. oxytoca, and P. mirabilis*) are unlikely to be at risk for increased *AmpC* expression (11, 14).

Despite relatively similar susceptibility percentages against Enterobacterales isolates, differences in PK/PD profiles should be considered when selecting among higher-generation oral cephalosporins (6, 8). More specifically, cefuroxime has an oral bioavailability of 30%–50% and protein binding of upwards of 50% (15). Cefpodoxime also has an oral bioavailability of up to 50%—depending on the dosage administered; however, significantly less protein binding (approximately 30%), allowing for higher drug concentrations in the bloodstream (16). Cefixime demonstrates an oral bioavailability of approximately 50% and protein binding of 65% (17). Cefuroxime, cefpodoxime, and cefixime may be able to reach PK/PD targets within the susceptible range when high, frequent dosages are administered to avoid subtherapeutic drug concentrations and after initial intravenous β-lactam administration (8). This is in contrast to cefdinir, which has an approximate 30% bioavailability and 70% protein binding—contributing to peak serum concentrations of less than 2.5 mg/L (18), making this agent less favorable for the treatment of GN-BSI (18). However, as oral cephalosporins are generally initiated after blood cultures have cleared and initial improvement in clinical response has been observed, some tradeoff between reduction in robust serum concentrations and improved quality of life may be acceptable (i.e., avoidance of a vascular catheter compared to intravenous beta-lactam antibiotics and fewer adverse events compared to oral fluoroquinolones and TMP-SMX).

Several limitations exist with this study. First, the number of isolates available for testing for a number of bacterial species was low (e.g., *Citrobacter koseri*), limiting inferences on species-specific susceptibility of oral cephalosporins. Additionally, although disk diffusion testing is considered a reference method for AST, variations exist between media and disks which may impact accuracy and reproducibility. Furthermore, triplicate testing to derive AST results was not performed which may have limited the accuracy of our findings. Logistical issues with obtaining all necessary agents precluded our ability to develop custom broth microdilution plates to derive MIC data for the oral cephalosporins tested.

Nonetheless, establishing ceftriaxone susceptibility as a reasonable proxy to infer susceptibility to higher general oral cephalosporins is the first step in considering the role of these agents in the treatment of GN-BSI. Additional investigations on their PK/PD and clinical effectiveness remain necessary. Overall, our findings indicate that ceftriaxone-susceptible isolates can serve as a reasonable surrogate for susceptibility to cefuroxime, cefdinir, cefpodoxime, and cefixime—particularly for common Enterobacterales species at low risk for AmpC production such as *E. coli*, *K. pneumoniae*, *K. oxytoca*, and *P. mirabilis*.
